# Promoting the health of refugee women: a scoping literature review incorporating the social ecological model

**DOI:** 10.1186/s12939-021-01387-5

**Published:** 2021-01-23

**Authors:** Maren M. Hawkins, Marin E. Schmitt, Comfort Tosin Adebayo, Jennifer Weitzel, Oluwatoyin Olukotun, Anastassia M. Christensen, Ashley M. Ruiz, Kelsey Gilman, Kyla Quigley, Anne Dressel, Lucy Mkandawire-Valhmu

**Affiliations:** 1 Joseph J. Zilber School of Public Health, University of Wisconsin - Milwaukee, Milwaukee, USA; 2 Department of Communication Studies, Towson University, Towson, USA; 3 College of Nursing, University of Wisconsin-Milwaukee, Milwaukee, USA; 4 Center for Advancing Population Science, Medical College of Wisconsin, Milwaukee, USA; 5 Master of Sustainable Peacebuilding, College of Nursing, University of Wisconsin-Milwaukee, Milwaukee, USA; 6 Jackson School of International Studies, Western Washington University, Bellingham, USA

**Keywords:** Literature review, Refugee women’s health, Health equity, Social ecological model

## Abstract

The health of refugee women after settlement in a new country, can be adversely or positively affected by individual, interpersonal, community, and organizational factors. While much of the previous literature highlights these factors individually, there is a lack of comprehensive synthesis regarding how the factors interact to influence the health of refugee women. We conducted a thematic analysis in our literature review to elucidate how providers can work with refugee women to prevent adverse health outcomes and intervene at multiple levels to improve their health outcomes after resettlement. We reviewed peer-reviewed literature from 2009 to 2019 from Google Scholar, JSTOR, Global Health, PubMed, CINAHL, Sociological Abstracts, and Social Service Abstracts, and also used citation chaining, to identify relevant information pertaining to refugee women’s health. The key terms used for our literature review were, health care, violence, social support, and mental health. In total, we included 52 articles, 3 books, and 8 other sources. We found that refugee women are vulnerable to violence during migration and typically have high rates of post-traumatic stress disorder. There were also concerns of secondary victimization by providers after resettlement. We also found that social support is an important factor for reducing isolation, and improving access to health care, as well as improving mental health outcomes. However, social support was often difficult to maintain, and was moderated by factors such as English language fluency. Health care was influenced by health literacy, cultural difference, communication concerns, and access issues. The findings suggest that at the individual and interpersonal levels there is a need to address language barriers, improve provider-patient communication, and provide appropriate medical and mental health screenings. At the organizational level, inter-organizational communication and awareness are vital. At the community level, providers can work with community leaders, to educate, create dialogue and collaboration, to help facilitate understanding and bolster community social support. Improved communication and knowledge about the unique needs and concerns of refugee women through an integrated, multi-system approach is necessary to improve their health outcomes.

## Introduction

Navigating healthcare systems and engaging in healthy behaviors can be difficult for those born in the countries they reside in; refugees however, contend with additional challenges and a myriad of factors affecting their health outcomes. In providing some context into what or who constitutes the refugee population, the United Nations High Commissioner for Refugees (UNHCR) describes a migrant as someone who left their home for a variety of reasons, including but not limited to, seeking better education, work, or reuniting with family; [1] while a refugee is someone who leaves due to “war, violence, conflict or persecution” [2]. Castañeda and Holmes, however, caution on the limitations of these definitions, as they argue “[w]hether a person is identified as a refugee or as some other socially constructed category... depends on historical, sociocultural, political, and economic contexts” [3]. There are nuances and implications for the use of the terms refugee versus migrants in regard to the choice to emigrate. These delineations are not necessarily rigid, [4] and the notion of choice is one that can be contested given the push and pull factors that cause people to emigrate, which for immigrants, are often economic, with important implications for health and well-being.

According to the UNHCR, the most recent data from 2018 indicated that there are over 25.9 million refugees worldwide, more than at any time in history [5]. As of 2015, the three countries that resettled the greatest number of refugees were, first, the U.S. who resettled 52,583 refugees, second, Canada who resettled 10,236 refugees, and third, Australia who resettled 5,211 refugees [6]. Following these three countries, there was Norway (fourth), Germany (fifth), Sweden (sixth), the United Kingdom (seventh), Finland (eighth), New Zealand (ninth), and France (tenth) [6]. The process of resettlement in another country is lengthy. In the U.S. for example, the process of resettlement takes at least two years and involves intensive medical and security screening by at least fifteen different agencies [7]. Over 3 million refugees have come to the U.S. since 1975 and resettled in all 50 states [7]. Once they arrive, refugee health screening, care, access, and clinical resources vary among states [8]. The official Domestic Medical Screening Guidelines Checklist by the Office of Refugee Resettlement (ORR) is strictly physical and assesses cholesterol, hepatitis, HIV/AIDS, and Tuberculosis (TB) status, but does not assess any psychosocial aspects of health [9]. The U.S. Centers for Disease Control and Prevention (CDC) has twelve additional recommended screening guidelines, including mental health, which are not mandatory [9]. Additionally, in Australia for example, from 2014 to 2015 there was an average waiting period of 14.5 months to receive a refugee visa [6]. Moreover, since 1977, Australia has been among the top three countries in the world for refugee resettlement [6]. Australia accepted the largest number of refugees from 1980 to 1981 with 20,795, and from 1975 to 1978, and then from 1984 to 2012 and 2013–2016, Australia accepted less than 10,000 refugees per year [6]. Resettlement in Australia also involves medical screening as well as character screening, which includes screening of “criminal conduct”[10], of the individual or family and family they may be reunifying with in the country of resettlement [10]. In countries of resettlement, health care and social service providers who work with refugees, particularly refugee women, need to be equipped to work in a culturally safe manner and to be sensitive to their unique needs.

The existing body of literature focused on aspects of refugee health, such as prenatal appointments or mental health among refugee women. While this research has been valuable for informing recommendations for best practices with this population, it lacked a holistic approach that includes other salient factors (e.g., social support, barriers in the healthcare system, and cultural realities) that impact women’s health, post-settlement. Literature on this topic tends to focus on one specific population and one specific health outcome among refugee women. For example, Marshall et al., focused solely on the mental health of Cambodian refugees ages 35–75 in their study [11]. In this article, we conduct a comprehensive synthesis of the literature on the topic through thematic review to identify social, cultural and environmental factors that affect refugee women’s health. While we examine the U.S.-specific context, the literature covers several different resettlement countries, including the U.S., such as Australia, Belgium, the Netherlands, South Korea, and the United Kingdom; thus, the findings have implications for other nations that resettle refugees. This is because, as we demonstrate in our review of the literature, many factors that influence refugee women’s health, such as patient-provider interactions and migration history, impact health regardless of the resettlement country. Furthermore, resettlement countries have different healthcare systems. For example, in Australia there is a mix of universal public and elective private health insurance, in the Netherlands the government provides most care through publicly financed health insurance, and in the U.S. while there are some programs for children and the elderly, most Americans have private health insurance and many are uninsured [12].

In our review we uncover how different ecological factors impact refugee women’s health post-settlement. Our key research questions were 1) what arenas, such as health care, social support, violence, and mental health, help or hinder refugee women’s health post resettlement, and 2) how can the Social-Ecological Model (SEM) (individual, interpersonal, organizational, and community levels) framework be adapted to inform implementation of programs targeting refugee women at various levels?

To address our research questions, we conducted a two-tier analysis of the literature. First, we conducted a deductive thematic analysis of the literature guided by a cultural safety lens. A cultural safety lens means that providers examine the power dynamic of their relationships with patients as well as participate in sensitive reflections of their interactions with patients.[13] Second, we created an adapted SEM model to provide clear framing and recommendations in our discussion section for improving the health of refugee women. This article presents information on the background of refugees as well as experiences with violence, mental health, health care and social support. Our methodology is fully explicated in the methods section.

### Positionality of the Authors

At the time of writing this article none of the authors were refugees themselves. The authors were graduate students, undergraduate students, and faculty in schools of Public Health, Nursing, and International Studies, in departments of Communication Studies, Sustainable Peacebuilding, Epidemiology, and Community and Behavioral Health Promotion. Most authors were at a large, public, urban university in the midwestern U.S. They identify as Latina, Black, Filipina, and White. Authors have served in the Peace Corps and AmeriCorps. They worked as nurses, including as a Sexual Assault Nurse Examiner, and three are African immigrant women who conducted research and work with immigrant and refugee populations in the U.S. While this is primarily a literature review, positioning ourselves at the time of our writing, and this article was key to respecting the agency of refugees and noting our own biases.

## Theoretical framework

In this manuscript we incorporate a lens of cultural safety (CS). CS is distinguished from other perspectives, namely cultural competence, because it requires healthcare providers to examine issues of power in their relationship to, and interactions with, patients who have been relegated to the margins of society [13]. In the context of CS, culture among refugee women is represented by the fluid and dynamic ways in which individuals identify at any given time, including but not limited to race, gender identity, sexual orientation, and socio-economic status. We incorporate CS in our review to highlight the need for sensitive reflection among providers serving refugee women.

### The social‐ecological Model

The social-ecological model (SEM) of health is crucial to framing this article. The CDC notes that in order to prevent and address adverse health outcomes, a multilevel approach is key.[14] The agency recognizes that, “[t]his model considers the complex interplay between individual, relationship, community, and societal factors, [and] allows for organization of the range of factors that put people at risk.” [14] SEM aids us in conceptualizing and understanding the individual, interpersonal, organizational, and community factors that impact health.

A key assumption of such ecological models is that in addition to individual characteristics, social and physical environments impact health, and both environments are dynamic and complex [15]. Another key assumption is that complex relationships, organizational, and community systems influence one another [15]. However, SEM is itself not a theory, nor does it describe the “variables of processes at each level expected to be the most influential on behavior” [16]. SEM is a framework and allows us to organize a comprehensive model of the factors influencing health within a population in order to thoughtfully inform interventions.[16] SEM is also used as an interpretive framework. For instance, Mengesha and colleagues, [17] used the framework to identify factors influencing access to health care among refugees and migrants in Australia. This is what we have done in this literature review.

We adapted SEM for the refugee context by drawing upon two ecological models for displaced persons and refugees, one by Keygnaert and colleagues [18] and another by Wells and colleagues [19] and based on our review of the literature (Table 1).

**Table 1:** Adapted Socioecological Model (SEM)

*Individual* -Migration history -Age -Income -Education -Knowledge about health care in the U.S. -Beliefs about healthcare	*Interpersonal* -Communication with healthcare provider -Role loss and interpersonal relationships -Changes to social networks -Violence, re-traumatization -Interpretation/ Translation services -Time spent with providers	*Organizational* -Relationships between organizations serving refugees -Links for interpretation and interpersonal services/relationships -Logistics of navigating systems (health insurance, transportation)	*Community* -Relationships and communication between refugee-serving organizations renegotiation -Stress and institutional racism

## Methods

We conducted a scoping literature review of the existing literature guided by the SEM. We restricted searches to Google Scholar, JSTOR, Global Health, PubMed, CINAHL, Sociological Abstracts, and Social Service Abstracts, to the years 2009–2019, and to works published in English. Prior to initiating the search, key themes were identified based on our research questions. These themes were: refugee health, refugee women’s health, violence, social support, health care access, and successful program models. The themes were then organized into three major categories: health care access, violence and mental health, and social support. The authors also used citation chaining to identify other relevant information and to cite the original sources of articles. We conducted our literature search by including the SEM term, but this yielded only three relevant results. We therefore broadened our search to include articles that did not explicitly address SEM, but to which the SEM framework could be applied and assessed. Each author reviewed literature on a specific area, such as violence faced by refugee women or health care access, and these articles were then screened by the lead author. We included peer-reviewed articles, such as literature reviews, qualitative studies, quantitative studies, and conceptual articles. Articles included both women and men and there was no limit on the age of refugees in the studies. We also included books, government websites, and one professional website for practicing nurses. We did not include non-peer reviewed dissertations, newspaper articles, or blog posts. Articles were screened by title and abstract before considering the whole article. After removing duplicates, a total of 96 full-text articles and 31 other sources were screened for eligibility. In the final analysis, we included 52 articles, 3 books, and 8 other sources.

## Results

Of the 52 articles reviewed, 37 focused on the health of refugee women. The remaining 15 articles provided were conceptual and provided supporting information on topics such as general information about health literacy among refugees. The 37 articles focused on a variety of populations, resettlement countries, and types of studies, such as literature reviews, qualitative and quantitative studies. Regarding the type of study, qualitative studies were most common (26 articles), and three of those 26 used a Community-Based Participatory Research approach. There were four quantitative studies. There were also five literature reviews, one meta-analysis, and one program evaluation.

Thirteen studies did not indicate the countries of origin of the refugees they worked with, including three of the four quantitative studies reviewed. Three articles each mentioned the region of origin, but not the country, such as Africa, Latin America, or South East Asia. However, Somalia was the most common country of origin, with six articles focusing on this group. This was followed by Cambodia (three articles), Myanmar (two articles), Syria (two articles), Bhutan (two articles), and the Democratic Republic of the Congo (two articles). Additional countries, such as Eritrea, Sudan, Vietnam, Afghanistan, Iraq, and Germany were mentioned in one article either as the sole population of interest or as part of a group.

There was significant variation in the country of origin in the articles reviewed. There was less variation regarding the country of resettlement. In 17 of the articles, the country of resettlement was the U.S. This was followed by Australia (six articles) and the United Kingdom (three articles). The other countries of resettlement were Jordan, Belgium, the Netherlands, South Korea, Sweden, Finland, and Germany. Only one article did not mention resettlement in a specific country, but stated that resettlement was in Europe.

Most of the articles focused solely on women (22 articles); including one article that included minor girls, and another one article that focused solely on elderly women. Three articles focused on elderly men and women, and 13 articles included both men and women of all ages. The four remaining articles were literature reviews in which there was no emphasis on gender.

The results are organized into subsections based on the major themes of violence and mental health, social support, and healthcare access.

## Violence and Mental Health

Limited data currently exist examining refugee women’s experiences of sexual assault (SA) and/or intimate partner violence (IPV) [20]. However, most adult refugees are women who consistently demonstrate high levels of trauma [11, 20, 21]. Young refugee women are vulnerable to experiences of SA and/or IPV during the migration process. They thus need reproductive health care during resettlement [20, 22]. One study in California found one in three refugee women reported having experienced a traumatic event prior to migration: 17 % physical assault, 11.7 % captivity, 10.5 % sexual assault, and 17.4 % weapon assault [20]. Although limited research exists examining SA and IPV among refugee women, the literature shows that “refugee women resettled in Western countries possess a tenfold risk of developing posttraumatic stress disorder (PTSD) symptoms” compared to non-refugee women [21].

Refugee women face unique challenges related to exposure to prior conflict-related trauma experienced before and during the migration process, as well as vulnerabilities related to resettlement [20, 21]. Hence, in an effort to provide culturally safe care, it is imperative that providers working with refugee women recognize this vulnerability and seek to prevent the occurrence of re-traumatization [21] that may be perpetrated by a husband or partner, [22] or even inadvertently by a provider through secondary victimization. Secondary victimization “refers to behaviors and attitudes of social service providers that are ‘victim-blaming’ and insensitive.”[23] Victimization by a provider is similar to victimization by a perpetrator in that both ignore the victim’s needs and devalue their experiences [23].

If the healthcare needs of refugee women who experienced SA and/or IPV are to be adequately addressed, it is essential that healthcare institutions provide flexible and comprehensive services that are capable of engaging refugee women as participants within their plan of care. Keygnaert and colleagues [18] conducted a qualitative study with migrants and refugees in Holland and Belgium, in which they found that healthcare, mental health services, and social services need to be readily available. Hence, services, such as rape crisis centers and IPV shelters, play vital roles and should be available. In the U.S., the Violence against Women Act stipulates payment for sexual assault exams and medical forensic exams for all survivors of sexual assault, including refugees [24]. These services are provided in collaboration with community-based advocacy organizations, which is considered best practice for survivors of sexual assault [24]. However, access to forensic exams may be difficult due to lack of trained nurse examiners, as well as lack of relevant programs [25].

## Social support

Social support refers to a tangible social network and the psychological benefits and resources available within the network to help individuals cope and improve their quality of life [26]. Social support is grouped into three main types: emotional, informational, and instrumental,[27] and comes from many avenues: family, friends, co-workers, religion, and the community. Refugee women acknowledge the importance of social support, but also face barriers to maintaining it [28]. Refugee women are frequently separated from family and friends, dispersed in unknown areas away from their communities, and sometimes cannot communicate in the host country’s language. Strong social support networks, particularly those developed shortly following resettlement, can improve access to healthcare services, reduce isolation, increase life satisfaction, mediate stress from discrimination, and protect against poor physical and mental health outcomes [29].

Length of time in the U.S. may play a role in social support. Kingsbury, et al., [30] examined factors associated with social support networks of pregnant Bhutanese refugee women, in Ohio. Women who had resettled from another city or state in the U.S. were more likely to report high levels of social support, which was mainly provided by family members and spouses, than women who had resettled directly from Bhutan.[30].

With approximately 25 % of women refugees being of reproductive age, [30] social support can be vital in pregnancy outcomes [30]. Women who have children or give birth shortly after migration face elevated levels of physical and emotional hardship; and social support tends to be lower as they often lack the family and community support in raising their children that would be present in their home country [31].

Social support can also play a major factor in parenting, as many women struggle to transition from a more interdependent culture in their country of origin with a large network of family and community support to a more independent culture in the U.S. that emphasizes autonomy and individualism [32]. Many refugee women want to keep issues within the family and are reluctant to reach out to healthcare facilities or individuals with a different background than themselves [33]. Moreover, many refugees contend with a loss of resources during migration, both physical and social, experience a renegotiation of their roles in their new communities, and cope with stress and institutional racism in the U.S. [19]. Social support from women of similar backgrounds and support from mothers in the host country, often through church groups or non-profit organizations, help women transition to the shift in culture, though social support among women from similar backgrounds seems to be cited by women more often than other sources [30]. English fluency also moderates the value of social support from the host community, meaning refugees who speak or understand English are more likely to engage better with the host country and thus experience enhanced social support compared to those who do not speak or understand English [34].

## Health Care

Migrating to a new country comes with anticipated and unforeseen challenges, [35] which include language barriers, [36] culturally discordant health beliefs, [37–40] issues with education access, [41] limited knowledge of the host culture, [42] and difficulties with healthcare access.

Healthcare access challenges include cultural differences regarding understanding of health care, [40] health insurance, [39] low health literacy, communication challenges, and in some cases racial discrimination. These challenges are more prominent, particularly for female refugees, as they intersect with other reproductive health needs that may significantly affect access to healthcare services [40, 43]. Given the circumstances surrounding their migration to the U.S., many refugee women have limited or no formal education [37, 40, 43, 44]. Moreover, their children are less likely to be in school; only 24 % of high school-age refugees are enrolled in high school and even fewer (3 %) continue their education past secondary school [5]. The disparity grows when it is disaggregated by sex, whereby, “[f]or every ten refugee boys in secondary school there are fewer than seven refugee girls”, [5] which has implications for health given that those with lower education levels have poor health outcomes [45]. In addition, education influences health literacy, and previous studies have found that health literacy mediates the relationship between education, health status, and health behaviors [46, 47].

The challenges facing refugees in the countries they resettle in are not strictly related to lack of healthcare support, as the U.S. and Germany, for example, have established programs that cater to the healthcare needs of refugees, at least for a limited period [48, 49]. There are deeper, structural issues that impact access to healthcare services by this population, including communication and cultural issues.

### Communication with Healthcare Providers

Communication is a major challenge for refugee women when accessing U.S. healthcare services [39, 44, 50, 51]. This challenge presents in various forms including limited English proficiency, limited health literacy, lack of interpretation services, and lack of interpersonal relationships [40]. In reporting the challenge of lack of interpersonal relationships, Carroll et al., found that refugee women from Somalia reported concerns about “depersonalized care, being rushed through the visit … and demonstrations of impatience or visible frustration due to language barriers on the part of healthcare providers or staff” [43]. This is an example of culturally unsafe care. Given the collectivist culture in many African countries, [52] the sample of women in the study had expectations of collegial interactions with their providers; however, their expectations were unmet as a result of the structure of the U.S. healthcare system (time-bound provider-patient interactions) and other challenges that made women feel disrespected and devalued because their providers did not display the level of interpersonal relationship they expected.

A lack of culturally-appropriate interpretation services is another dimension of communication challenges. As mentioned earlier, reproductive health is a salient part of women’s lives, especially among many refugee women. Discussing issues of reproductive health with healthcare providers can be particularly challenging for refugee women [37]. For instance, in another study with Somali refugee women, [37] female circumcision, a cultural reproductive health practice for many Somali women (but an illegal practice in the U.S.), was considered to be a challenge when interacting with healthcare providers because many were unaware of this practice. Female circumcision can be an emotionally charged subject and healthcare providers should avoid imparting judgement, stereotyping, and stigmatizing women who have undergone this procedure [53]. This is where CS can inform healthcare practice. Critical reflection on personal feelings and bias around this practice needs to take place prior to working with the patient. The healthcare provider should be prepared to preserve the patient’s sense of dignity and provide appropriate health care when the situation arises.

Language barriers versus access to culturally appropriate interpretation services are reported to be another major challenge for refugee women. In a qualitative study, Mirza et al., found that interpretation services come with challenges such as finding trained interpreters who can understand the multiple languages that refugees (even those from the same country) speak [39]. These communication challenges are a barrier to refugees accessing healthcare services [39]. Literacy and language challenges hinder the promotion of health equity for refugees accessing healthcare. Addressing the challenges of lack of interpreters, cultural differences in health care, and institutional racism, requires changes at the interpersonal point of healthcare access as well as at the organizational and societal levels. This may include allowing for additional time for provider-patient interactions in order to make accommodations for relationship building, addressing language barriers and unique health education needs. Health educators also need to be adequately educated and informed about the various healthcare beliefs, practices, and needs of the refugee populations with whom they interact.

Refugees face barriers when navigating complex systems such as health care and health insurance, [54] including access to transportation, complexities of health insurance plans, and the cost of health care [55]. Logistical issues, such as scheduling, hours of services, wait times for appointments, child care during appointments, and transportation are recurrent challenges [55]. Issues around insurance enrollment or lack of insurance through an employer create additional gaps with healthcare cost and insurance access [55]. These are community and organization-level issues that lead to avoidable consequences, such as missing appointments or lacking insurance.

Reavy et al. [56] proposed a new clinic model for addressing the barriers to accessing healthcare among refugees through a case study that utilized an ecological care model named C.A.R.E. (Culturally Appropriate Resources and Education). This model drew upon the theory of cultural safety [56]. The nurse-led clinical program provided a smooth continuum of care, as well as education specific to refugee patients and family well-being, which was taught by a Clinical Health Advisor in a group setting. The model considered power dynamics, bridged the gap between providers and refugees, built a relationship based on shared perspectives provided by a Clinical Health Advisor who was also of the same background as the refugee, and thus provided additional lived experience peer support [56].

The Clinical Health Advisor may encounter challenges related to lack of communication with healthcare providers since they are not Certified Medical Interpreters privileged to know patient or visit information [56]. However, health education programming, involving the use of Clinical Health Advisor, may bridge the gap in understanding between refugees and providers and thus contribute to positive impact on health outcomes and reduction in disparities [57]. Mancuso highlighted the importance of considering how health care, culture, and social setting are interconnected, and suggested a strengths-based, bidirectional approach to community outreach and programming focused on eliminating barriers by leveraging refugee community strengths [57]. Further, Mancuso stressed the importance of building trust, communication, and collaboration in order to effectively engage with the healthcare system [57].

## Discussion

Refugee women are a vulnerable population, with unique needs requiring particular attention, of which their providers need to be aware [58]. As previously noted, while SEM is not a theory and cannot tell us about the relationship between constructs ,[16] SEM does help us conceptualize the multilevel factors that impact health, adhering to the assumption of ecological models that the interaction between the levels is dynamic [14, 15]. What this means is that while we cannot explicate the relationships between variables as it is outside the scope of SEM in this article, we need to remember that these levels do influence one another and interventions that are multilevel tend to be more effective for that reason [16]. Our review of the literature shows that factors that perpetuate health inequities among refugee women include individual, interpersonal, organizational, and community factors, and hence different levels necessitate different recommendations.

### Recommendations according to the adapted SEM framework

#### Individual and interpersonal levels

The individual and interpersonal levels within the adapted SEM framework are discussed together because addressing individual factors involves interpersonal interactions. For example, a refugee woman with minimal formal education is at risk of poor health outcomes due to potentially low health literacy. However, to improve the level of health education, this involves interpersonal communication with a provider.

Migration history, age, level of education, knowledge about services, and beliefs about health all shape an individual’s susceptibility and resilience to poor health during migration and post-settlement [5, 18, 20, 37, 40, 43, 44, 54]. For example, a low level of formal education and a difficult migration history have the potential to lead to poor health status and health behaviors [21, 46, 47]. Communication with providers and appropriate screenings and referrals are therefore important for promoting health equity among refugee women at the individual and interpersonal levels. While healthcare providers are not expected to offer formal education, improved communication, through greater time, dialogue, and clarity of medical questions and instructions may help to bridge gaps in health literacy. Hence, those providing services to refugee women should be privy to and ask about the individual’s experiences and preferences when receiving care. Asking about preferences and how one would like to be treated is a way of conferring some choice to the refugee. Moreover, mental health screening, particularly perinatal and PTSD screening is vital for refugee women [59]. While mental health screening forms exist, this screening is optional [9]. Providers should incorporate these screenings as part of standard procedure. Additionally, evidence shows that poor social support among refugees contributes to poor well-being, which suggests a need for screening for social support [59].

Part of interpersonal communication between providers and refugees should emphasize making women aware of available services. A survey of refugee women coping with postpartum depression found many were unaware of services available to them, but once aware, they generally found the services useful [60]. In addition, many services were different from what was available in their country of origin, thus women may not even know what to look for [60]. Consistent with other studies, positive relationships with providers and social support from family, friends, and intimate partners increases the likelihood of seeking, accepting, and/or continuing care [60].

Providing culturally safe care by critically analyzing how social, political, and historical factors may impact the health of refugee clients, based on evidence, is critical in order to deliver care that does not demean, re-traumatize, or further marginalize refugee populations. By developing a deep understanding and acknowledgement of how multilevel, structural factors such as racism and provider-client power differentials impact the lived experiences of refugees, both within their healthcare encounters and society at large, providers can then engage in critical reflexivity on how their practices and beliefs relative to refugee clients are shaped by the same structural factors.

### Organizational level

It is crucial that healthcare providers are aware of other organizations serving refugees. Compiling a list of local organizations serving refugees and the services they provide, and providing this list to patients would help streamline connection to services. Additionally, to improve transition in care, the use of volunteers to aid with healthcare system navigation is an effective approach [61].

### Community Level

Providers serving refugees can educate faith and community leaders on the psychological stressors faced by refugee women, and encourage compassion, confidentiality, and regular contact. This can be beneficial in increasing resources for social support for women as well as in identifying women at risk [62]. Identifying local organizations and community representatives and engaging them in determining ways to better serve women could be a valuable approach. In addition, when women present themselves at a healthcare facility, providers have the responsibility to offer compassionate, culturally humble, and collaborative care. Language also tends to be a major barrier to accessing social support in the host community. Encouraging involvement in English language courses, [34] or increasing availability of interpreters, facilitates further social support and helps ease the transition.

## Limitations

This article did not consider a comprehensive review of discrimination, and violence, particularly sexual violence, faced by refugees. Racial discrimination is briefly mentioned as it relates to service provision; however, further elaboration was beyond the scope of this article, but is an important consideration for future research to contribute to filling the gap in the literature on the topic. We also recognize that each refugee and each group of refugees may have vastly different needs and that this article is a synthesis of the literature as it relates to all refugee women, which may be a limitation to identifying the needs of specific groups. Moreover, much of the literature combined immigrants and refugees. Thus, it was in some cases difficult to ascertain evidence specific to refugees. Additionally, this paper lacks specific evidence for older adult refugee women. There is limited research on the experiences of older adult refugee women and their unique experiences. Aging refugees have often resided in the U.S. for years or decades, [63] and not much is known about their needs and challenges following their immediate resettlement [63–67]. Furthermore, the studies cited in this article have varying study designs and sample sizes, many of which were small, which may affect the interpretation and generalizability of our findings.

## Conclusions

Violence, lack of social support, and limited access to services can negatively impact the health of refugees. Thus, promoting the health of refugee women in order to enhance health equity by preventing and reducing avoidable and unjust outcomes requires a multilevel and multisystem approach. Social support, culturally safe providers, and patient navigators (e.g., volunteers in the healthcare system, or clinical health advisors), can increase refugees’ comfort, knowledge, participation, and trust in the health system. Improved communication between organizations can streamline care and reduce barriers to accessing services; while greater recognition of the changed roles and resource loss that many refugee women face, and working with the community to devise solutions, can improve the health of refugee women.

## Data Availability

Not applicable.
